# Supplementary material for: Thermal proteome profiling unveils protein targets of deoxycholic acid in living neuronal cells
Source: Adv Biotechnol (Singap). 2023 Dec 12;1(4):7. doi: 10.1007/s44307-023-00007-3 (PMC11727579; doi:10.1007/s44307-023-00007-3)
Supplement: Supplementary file 1 — Additional file 1: Figure S1. The melting curve of the SH-SY5Y lysate treated with DCA and DMSO (control) treatment. 53°C was regarded as the median protein thermal melting temperature (Tm). Table S1. the list of the 65 DCA-interacting proteins. [file 44307_2023_7_MOESM1_ESM.docx]

**Thermal Proteome Profiling Unveils Protein Targets of Deoxycholic Acid in Living Neuronal Cells**

Hemi Luan^1#*^, Xuan Li^2#^, Wenyong Zhang^2^, Tiangang Luan^1,3,4*^

1.Department of Biomedical Engineering, School of Biomedical and Pharmaceutical Sciences, Guangdong University of Technology, Guangzhou, 510006, Guangdong, China

2. School of Medicine, Southern University of Science and Technology, Shenzhen, China

3. Institute of Environmental and Ecological Engineering, Guangdong University of Technology, Guangzhou 510006, China.

4. School of Biotechnology and Health Sciences, Wuyi University, Jiangmen 529020, China

# These authors contributed equally to this work.

***Correspondence**

Prof. Hemi Luan

Email : [hm-luan@msn.com](mailto:hm-luan@msn.com)

School of Biomedical and Pharmaceutical Sciences, Guangdong University of Technology, China

Prof. Tiangang Luan

Email : [cesltg@mail.sysu.edu.cn](mailto:cesltg@mail.sysu.edu.cn)

School of Biotechnology and Health Sciences, Wuyi University, China


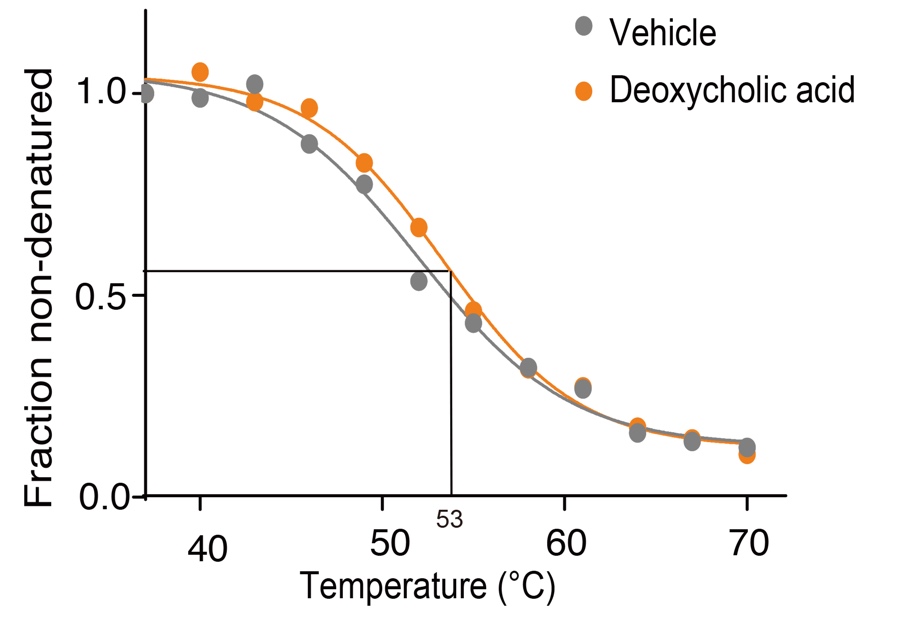


**Figure S1.** The melting curve of the SH-SY5Y lysate treated with DCA and DMSO (control) treatment. 53°C was regarded as the median protein thermal melting temperature (Tm).

| Table S1 the list of the 65 DCA-interacting proteins | |
| --- | --- |
| Accession | Description |
| **P13667** | Protein disulfide-isomerase A4 OS=Homo sapiens OX=9606 GN=PDIA4 PE=1 SV=2 |
| **P30101** | Protein disulfide-isomerase A3 OS=Homo sapiens OX=9606 GN=PDIA3 PE=1 SV=4 |
| **Q14697** | Neutral alpha-glucosidase AB OS=Homo sapiens OX=9606 GN=GANAB PE=1 SV=3 |
| **Q99714** | 3-hydroxyacyl-CoA dehydrogenase type-2 OS=Homo sapiens OX=9606 GN=HSD17B10 PE=1 SV=3 |
| **P20700** | Lamin-B1 OS=Homo sapiens OX=9606 GN=LMNB1 PE=1 SV=2 |
| **P23368** | NAD-dependent malic enzyme, mitochondrial OS=Homo sapiens OX=9606 GN=ME2 PE=1 SV=1 |
| **Q16352** | Alpha-internexin OS=Homo sapiens OX=9606 GN=INA PE=1 SV=2 |
| **P23246** | Splicing factor, proline- and glutamine-rich OS=Homo sapiens OX=9606 GN=SFPQ PE=1 SV=2 |
| **Q16836** | Hydroxyacyl-coenzyme A dehydrogenase, mitochondrial OS=Homo sapiens OX=9606 GN=HADH PE=1 SV=3 |
| **Q53EL6** | Programmed cell death protein 4 OS=Homo sapiens OX=9606 GN=PDCD4 PE=1 SV=2 |
| **Q03252** | Lamin-B2 OS=Homo sapiens OX=9606 GN=LMNB2 PE=1 SV=4 |
| **O95302** | Peptidyl-prolyl cis-trans isomerase FKBP9 OS=Homo sapiens OX=9606 GN=FKBP9 PE=1 SV=2 |
| **Q16799** | Reticulon-1 OS=Homo sapiens OX=9606 GN=RTN1 PE=1 SV=1 |
| **Q02809** | Procollagen-lysine,2-oxoglutarate 5-dioxygenase 1 OS=Homo sapiens OX=9606 GN=PLOD1 PE=1 SV=2 |
| **Q9H307** | Pinin OS=Homo sapiens OX=9606 GN=PNN PE=1 SV=5 |
| **E7ET15** | U2 snRNP-associated SURP motif-containing protein OS=Homo sapiens OX=9606 GN=U2SURP PE=1 SV=1 |
| **Q96I25** | Splicing factor 45 OS=Homo sapiens OX=9606 GN=RBM17 PE=1 SV=1 |
| **Q9UEY8** | Gamma-adducin OS=Homo sapiens OX=9606 GN=ADD3 PE=1 SV=1 |
| **Q86YP4** | Transcriptional repressor p66-alpha OS=Homo sapiens OX=9606 GN=GATAD2A PE=1 SV=1 |
| **Q9UKV3** | Apoptotic chromatin condensation inducer in the nucleus OS=Homo sapiens OX=9606 GN=ACIN1 PE=1 SV=2 |
| **E9PS76** | Probable RNA-binding protein EIF1AD (Fragment) OS=Homo sapiens OX=9606 GN=EIF1AD PE=1 SV=1 |
| **Q8IV36** | Protein HID1 OS=Homo sapiens OX=9606 GN=HID1 PE=1 SV=1 |
| **Q9Y305** | Acyl-coenzyme A thioesterase 9, mitochondrial OS=Homo sapiens OX=9606 GN=ACOT9 PE=1 SV=2 |
| **A0A2R8YEL6** | Glutamate--cysteine ligase catalytic subunit (Fragment) OS=Homo sapiens OX=9606 GN=GCLC PE=1 SV=1 |
| **Q14696** | LRP chaperone MESD OS=Homo sapiens OX=9606 GN=MESD PE=1 SV=2 |
| **Q92542** | Nicastrin OS=Homo sapiens OX=9606 GN=NCSTN PE=1 SV=2 |
| **Q12907** | Vesicular integral-membrane protein VIP36 OS=Homo sapiens OX=9606 GN=LMAN2 PE=1 SV=1 |
| **E9PQ61** | Zinc finger CCCH domain-containing protein 11A OS=Homo sapiens OX=9606 GN=ZC3H11A PE=1 SV=1 |
| **Q9C0E8** | Endoplasmic reticulum junction formation protein lunapark OS=Homo sapiens OX=9606 GN=LNPK PE=1 SV=2 |
| **Q14118** | Dystroglycan OS=Homo sapiens OX=9606 GN=DAG1 PE=1 SV=2 |
| **P57737** | Coronin-7 OS=Homo sapiens OX=9606 GN=CORO7 PE=1 SV=2 |
| **Q92859** | Neogenin OS=Homo sapiens OX=9606 GN=NEO1 PE=1 SV=2 |
| **P48507** | Glutamate--cysteine ligase regulatory subunit OS=Homo sapiens OX=9606 GN=GCLM PE=1 SV=1 |
| **P50750** | Cyclin-dependent kinase 9 OS=Homo sapiens OX=9606 GN=CDK9 PE=1 SV=3 |
| **H0YEP5** | Sphingomyelin phosphodiesterase (Fragment) OS=Homo sapiens OX=9606 GN=SMPD1 PE=1 SV=1 |
| **Q32P28** | Prolyl 3-hydroxylase 1 OS=Homo sapiens OX=9606 GN=P3H1 PE=1 SV=2 |
| **Q15645** | Pachytene checkpoint protein 2 homolog OS=Homo sapiens OX=9606 GN=TRIP13 PE=1 SV=2 |
| **Q9NYF8** | Bcl-2-associated transcription factor 1 OS=Homo sapiens OX=9606 GN=BCLAF1 PE=1 SV=2 |
| **A0A087X1V5** | Nibrin (Fragment) OS=Homo sapiens OX=9606 GN=NBN PE=1 SV=1 |
| **O60831** | PRA1 family protein 2 OS=Homo sapiens OX=9606 GN=PRAF2 PE=1 SV=1 |
| **A0A087X0N3** | Nucleolar protein 4-like OS=Homo sapiens OX=9606 GN=NOL4L PE=1 SV=1 |
| **Q96A49** | Synapse-associated protein 1 OS=Homo sapiens OX=9606 GN=SYAP1 PE=1 SV=1 |
| **Q92600** | CCR4-NOT transcription complex subunit 9 OS=Homo sapiens OX=9606 GN=CNOT9 PE=1 SV=1 |
| **Q08174** | Protocadherin-1 OS=Homo sapiens OX=9606 GN=PCDH1 PE=1 SV=2 |
| **O14657** | Torsin-1B OS=Homo sapiens OX=9606 GN=TOR1B PE=1 SV=2 |
| **P09110** | 3-ketoacyl-CoA thiolase, peroxisomal OS=Homo sapiens OX=9606 GN=ACAA1 PE=1 SV=2 |
| **Q96KG9** | N-terminal kinase-like protein OS=Homo sapiens OX=9606 GN=SCYL1 PE=1 SV=1 |
| **Q9BQ39** | ATP-dependent RNA helicase DDX50 OS=Homo sapiens OX=9606 GN=DDX50 PE=1 SV=1 |
| **C9JDR0** | Sterol-4-alpha-carboxylate 3-dehydrogenase, decarboxylating (Fragment) OS=Homo sapiens OX=9606 GN=NSDHL PE=1 SV=1 |
| **O43150** | Arf-GAP with SH3 domain, ANK repeat and PH domain-containing protein 2 OS=Homo sapiens OX=9606 GN=ASAP2 PE=1 SV=3 |
| **Q9NZJ6** | Ubiquinone biosynthesis O-methyltransferase, mitochondrial OS=Homo sapiens OX=9606 GN=COQ3 PE=1 SV=3 |
| **Q13257** | Mitotic spindle assembly checkpoint protein MAD2A OS=Homo sapiens OX=9606 GN=MAD2L1 PE=1 SV=1 |
| **E2QRG8** | Receptor expression-enhancing protein (Fragment) OS=Homo sapiens OX=9606 GN=REEP5 PE=1 SV=1 |
| **Q9UJY4** | ADP-ribosylation factor-binding protein GGA2 OS=Homo sapiens OX=9606 GN=GGA2 PE=1 SV=3 |
| **Q9BU61** | NADH dehydrogenase [ubiquinone] 1 alpha subcomplex assembly factor 3 OS=Homo sapiens OX=9606 GN=NDUFAF3 PE=1 SV=1 |
| **Q96S59** | Ran-binding protein 9 OS=Homo sapiens OX=9606 GN=RANBP9 PE=1 SV=1 |
| **Q86V48** | Leucine zipper protein 1 OS=Homo sapiens OX=9606 GN=LUZP1 PE=1 SV=2 |
| **Q8NBX0** | Saccharopine dehydrogenase-like oxidoreductase OS=Homo sapiens OX=9606 GN=SCCPDH PE=1 SV=1 |
| **P11279** | Lysosome-associated membrane glycoprotein 1 OS=Homo sapiens OX=9606 GN=LAMP1 PE=1 SV=3 |
| **H0Y5S9** | Casein kinase I isoform epsilon (Fragment) OS=Homo sapiens OX=9606 GN=CSNK1E PE=1 SV=1 |
| **G3V502** | Mitochondrial import inner membrane translocase subunit Tim9 OS=Homo sapiens OX=9606 GN=TIMM9 PE=1 SV=1 |
| **A0A3B3IRM4** | Xylulose kinase OS=Homo sapiens OX=9606 GN=XYLB PE=1 SV=1 |
| **P51878** | Caspase-5 OS=Homo sapiens OX=9606 GN=CASP5 PE=1 SV=3 |
| **Q9P2X0** | Dolichol-phosphate mannosyltransferase subunit 3 OS=Homo sapiens OX=9606 GN=DPM3 PE=1 SV=2 |
| **Q8NCQ2** | Uncharacterized protein CSNK1G2-AS1 OS=Homo sapiens OX=9606 GN=CSNK1G2-AS1 PE=2 SV=2 |
